# Sexual function after total laparoscopic hysterectomy or transabdominal hysterectomy for benign uterine disorders: a retrospective cohort

**DOI:** 10.1590/1414-431X20199058

**Published:** 2020-02-14

**Authors:** Yiqun Wang, Xiaoyan Ying

**Affiliations:** 1 Jiangsu Zhenjiang Maternal and Child Health Hospital, Zhenjiang, China; 2 Second Affiliated Hospital of Nanjing Medical University, Nanjing, China

**Keywords:** Hysterectomy, Laparoscopy, Laparotomy, Sexual activity

## Abstract

The objective of this study was to evaluate changes in sexual function after total laparoscopic hysterectomy (TLH) or transabdominal hysterectomy (TAH). This retrospective cohort study included patients with benign uterine tumors that were divided into TLH group and TAH group based on the hysterectomy technique used. Baseline, intraoperative, and postoperative characteristics were compared between groups. Postoperative sexual function was assessed using the Brief Index of Sexual Functioning for Women. The TLH and TAH groups contained 119 patients (age, 51.5±6.1 years) and 126 patients (age, 50.0±4.7 years), respectively. Baseline characteristics were comparable between groups, although uterine size was larger in the TAH group (P<0.001). Compared with the TAH group, the TLH group had a longer operative time (130.0±36.2 *vs* 107.3±28.5 min, P<0.001), lower pain index at 24 h (2.0±1.6 *vs* 4.0±2.6, P<0.001), and shorter hospitalization time (5.7±1.1 *vs* 8.1±1.2 days, P<0.001). Many patients in the TLH and TAH groups reported decreased satisfaction with their sexual life (67.5 and 56.0%, respectively), reduced frequency of sexual activity (70.1 and 56.0%, respectively), decreased libido (67.5 and 56.0%, respectively), orgasm dysfunction (42.9 and 42.9%, respectively), and increased dyspareunia (77.9 and 85.7%, respectively). However, there was no significant difference between groups in any of the indexes of postoperative sexual function (P>0.05). Both TLH and TAH had comparable negative effects on sexual function in women treated for benign uterine tumors in China, with a decreased frequency of sexual activity, reduced libido, orgasm dysfunction, and increased dyspareunia.

## Introduction

Hysterectomy is the most common gynecologic surgical procedure performed worldwide, with rates of 2–5 per 1000 women in North America, Europe, Australia, and China (1–4). Hysterectomy is used to treat a wide variety of benign and malignant medical conditions, including fibroids, prolapse, abnormal uterine bleeding, pelvic pain, and cervical, endometrial, and ovarian cancer (5,6). Currently, hysterectomy is performed using one of three surgical approaches: laparoscopic, transabdominal, or transvaginal (7).

Although hysterectomy is generally considered to be a safe procedure, it is associated with a number of complications including vaginal cuff dehiscence and injuries to nervous, urinary, and gastrointestinal structures (7). There is also evidence that hysterectomy can lead to changes in sexual function, with differing effects depending on whether the underlying condition being treated is benign or malignant (8,9). Most studies have reported that hysterectomy for benign diseases results in an overall improvement in sexual function (10–15), likely because of the alleviation of symptoms such as abnormal uterine bleeding and pelvic pain (16). However, around 20–40% of patients with benign disease experience a worsening of sexual function after hysterectomy (17–19), and one study even showed an overall worsening of sexual function (20). Furthermore, clinical studies of women with malignant disease have consistently reported a worsening of sexual function after hysterectomy (21–26), and hysterectomy was found to be a factor associated with poorer sexual function in middle-aged women (27). Several studies have evaluated whether sexual function outcomes after hysterectomy are influenced by the technique used (9). However, limited data are available comparing sexual function outcomes between laparoscopic hysterectomy and transabdominal hysterectomy.

Therefore, the aim of this retrospective cohort was to compare the changes in the patients' sexual function between laparoscopic hysterectomy and abdominal hysterectomy.

## Material and Methods

### Study design and participants

This was a retrospective cohort of patients with benign tumors of the uterus admitted to Zhenjiang Maternal and Child Health Hospital, Jiangsu Province, China between January 2013 and December 2018. The inclusion criteria were: 1) menorrhagia, anemia, symptoms related to compression of adjacent organs, and/or progressive dysmenorrhea; 2) gynecologic and imaging investigations performed preoperatively and diagnosed as having cervical intraepithelial neoplasia (CIN) grade III lesions by a cervical human papillomavirus test, thin-prep cytologic test, colposcopy and biopsy, and/or loop electrosurgical excision procedure; and 3) no fertility requirements. The exclusion criteria to avoid complications affecting the operation effect and operation time were as follows: 1) surgical complications such as heart disease, thyroid and other medical diseases, and some surgical diseases such as extraperitoneal or sigmoid colon tumors; 2) cervical malignant lesions; 3) gynecologic malignant tumors; and 4) unstable clinical condition, such as blood pressure instability and uncontrolled blood sugar.

According to different operation methods, the patients were divided into total laparoscopic hysterectomy (TLH) and transabdominal hysterectomy (TAH) groups based on the hysterectomy technique used. The Medical Ethics Committee of Zhenjiang Maternal and Child Health Hospital approved the study. All patients provided informed written consent for their treatment.

### Surgical procedures

Patients underwent preoperative ultrasonography or pelvic magnetic resonance imaging, if necessary. Intravenous antibiotics were administered half an hour before surgery to prevent infection, and additional antibiotics were given if surgery lasted for more than 3 h.

#### TLH

After the successful induction of general anesthesia, a puncture outfit was inserted into the abdomen via an umbilical incision, and the abdominal cavity was insufflated with CO_2_ gas. A trocar was inserted into the abdomen at the umbilical incision, and a laparoscope was introduced. The adnexal area on both sides was exposed using a cup-type uterine manipulator. A vascular closure device was used for electrocoagulation during the approach to the uterine edge. The ovarian proper ligament, uterine round ligament, and broad ligament were cut, first on the right side and then on the left side. The reflected peritoneum of the uterus and bladder was opened with a vascular closure device. The bladder was pushed down to reach the lower part of the uterine manipulator, and any adhesions between the bladder and uterus were noted (to avoid inadvertent damage to the bladder). The para-uterine tissue was detached by blunt and sharp separation to expose the uterine blood vessels. The right and left uterine arteries and veins were cut and coagulated at the level of the uterine isthmus close to the uterine wall, with any hemorrhage controlled with electrocoagulation. The main body of the uterus and patellar ligament was cut using bipolar electrocoagulation, and the uterine fornix was cut along the ring of the uterine manipulator. The uterus was resected and removed through the vagina. The vaginal stump was continuously sutured with no. 1 absorbable surgical sutures under laparoscopy. The pelvic cavity was washed with saline and checked for any bleeding points. The trocar was removed, the small incision was sutured, and the specimens were sent for pathologic examination.

#### TAH

Following the induction of general anesthesia, a 10-cm longitudinal incision was made in the abdomen. If the patient already had a scar from previous pelvic surgery, the incision was made along the old scar. The uterus was exposed by separation of the abdominal tissues layer by layer. The uterus was lifted with curved forceps, and the uterine horns were clamped. The left round ligament was clamped, cut, and double-sutured with a no. 7 silk suture, and the same procedure was then applied to the right side. The left fallopian tube isthmus and ovarian proper ligament were clamped, ligated, and double-sutured with no. 7 silk suture, and the contralateral side was treated by the same method. The reflected peritoneum of the uterus and bladder was opened, and the bladder was pushed down to reach the lower part of the uterine manipulator, with care taken to avoid damage to the bladder. The uterine isthmuses were clamped, and the uterine arteries and veins were cut and double-sutured with no. 7 silk suture. The bilateral primary sacral ligaments and parametrial tissues were cut and clamped step-by-step, and the stump to the level of the external os of the cervix was ligated and sutured with no. 7 silk suture. The vaginal fornix was circumcised at the external os of the cervix. A piece of iodophor gauze was inserted into the vagina, and the uterus was removed. The vaginal stump was disinfected with iodophor and continuously sutured with 1–0 absorbable suture. After treatment of any active bleeding points and checking for any remaining intraabdominal gauze, the abdomen was closed layer by layer. After the operation, the intravaginal iodophor gauze was taken out, and the specimen was sent for pathologic examination.

### Outcome measures

Baseline demographic and clinical characteristics were extracted from the medical records. The operation time (from the start of the skin incision to the completion of skin incision suturing), volume of intraoperative blood loss, change in hemoglobin (Hb) level during surgery, pain index at 24 h after surgery, postoperative intestinal air exhausting time, postoperative hospital stay, and any postoperative complications were noted. Patients were followed-up by telephone. Postoperative sexual function, including time to recovery of sexual life, frequency of sexual activity, postoperative libido, postoperative orgasm, postoperative sexual intercourse disorder, and postoperative dyspareunia, was assessed using the Brief Index of Sexual Functioning for Women (BISF-W) (28). Each patient was followed up twice by telephone, and the interval was one month. The first follow up began more than half a year after the operation.

### Statistical analysis

The data were analyzed using SPSS 20.0 (IBM Corp., USA). Count data are reported as number (%) and were compared between groups using the χ^2^ test. Normally distributed continuous measurement data are reported as means±SD and were compared between groups using Student's *t*-test. P<0.05 was considered statistically significant.

## Results

### Demographic and clinical characteristics of the study participants

A total of 245 patients were included in the final analysis, with 119 patients (mean age, 51.5±6.1 years) in the TLH group and 126 patients (mean age, 50.0±4.7 years) in the TAH group. The main diagnoses were uterine fibroids, adenomyosis, uterine fibroids with adenomyosis, and CIN III lesions. There were no significant differences in baseline clinical characteristics between the two groups (Table 1), except that uterine size was significantly larger in the TAH group than in the TLH group (P<0.001).

**Table 1:** Comparison of baseline clinical characteristics between the total laparoscopic hysterectomy (TLH) and transabdominal hysterectomy (TAH) groups.

Characteristic	TLH (n=119)	TAH (n=126)	P
Age (years)	51.5±6.1	50.0±4.7	0.463
Marriage duration (years)	28.0±7.4	27.1±5.4	0.301
Body mass index (kg/m^2^)	24.3±3.0	24.3±2.9	0.987
History of pelvic surgery	37 (31.1%)	46 (36.5%)	0.371
History of vaginal delivery	105 (88.2%)	104 (82.5%)	0.208
Uterine size (gestational week)	8.7±3.7	11.1±3.1	<0.001
Preoperative hemoglobin (g/L)	117.3±20.3	112.9±21.3	0.099

Data are reported as means±SD or n (%). Statistical analysis was performed with Student’s *t*-test or the chi-squared test.

### Intraoperative and postoperative characteristics

The operative time was significantly longer for the TLH group than for the TAH group (P<0.001), although intraoperative blood loss was similar between groups (Table 2). Compared with the TLH group, the TAH group had a higher postoperative pain index at 24 h (P<0.001), longer postoperative air exhausting time (P<0.05), and longer postoperative hospital stay (P<0.001). Postoperative complications occurred in 10 patients in the TLH group (upper respiratory tract infection, n=4; postoperative fever, n=2; urinary tract infection, n=2; acute enteritis, n=1; liver dysfunction, n=1) and 9 patients in the TAH group (postoperative fever, n=2; secondary suture of the incision, n=2; upper respiratory tract infection, n=1; urinary retention, n=1; urinary tract infection, n=1; acute lumbar disc herniation, n=1; diarrhea, n=1). The incidence of postoperative complications did not differ significantly between groups, and all complications were symptomatically treated.

**Table 2:** Comparison of intraoperative and postoperative characteristics between groups.

	TLH (n=119)	TAH (n=126)	P
Operative time (min)	130.0±36.2	107.3±28.5	<0.001
Operative blood loss (mL)	95.2±68.7	113.0±89.3	0.083
Pain index at 24 h postoperatively	2.0±1.6	4.0±2.6	<0.001
Postoperative air exhausting time (d)	2.0±0.5	2.1±0.6	0.044
Postoperative Hb change (g/L)	9.1±9.1	8.1±8.2	0.387
Postoperative hospital stay (d)	5.7±1.1	8.1±1.2	<0.001
Postoperative complications	10 (8.4%)	9 (7.1%)	0.712

Data are reported as means±SD or n (%). Statistical analysis was performed with Student’s *t*-test or the chi-squared test. Hb: hemoglobin; TAH: total transabdominal hysterectomy; TLH: total laparoscopic hysterectomy.

### Evaluation of sexual function during follow-up

A total of 34 (28.6%) patients in the TLH group and 31 (24.6%) patients in the TAH group were lost to follow-up, with no significant difference between groups in the number lost to follow-up. Therefore, evaluation of sexual function was performed for 85 patients in the TLH group and 95 patients in the TAH group. The educational backgrounds of the patients and their spouses were similar (Table 3), indicating that this would not be a factor biasing the assessment of sexual function. The majority of patients in both groups resumed sexual activity, and there were no significant differences between groups in the proportion of patients resuming sexual activity or the time to resumption of sexual activity (P>0.05) (Table 4). After surgery, many patients in both groups reported decreased satisfaction with their sexual life (Table 5), a reduced frequency of sexual activity, decreased libido, orgasm dysfunction, and increased dyspareunia. However, there was no significant difference between groups in any of the indexes of sexual function (P>0.05).

**Table 3:** Comparison of the educational backgrounds of the patients and their spouses between groups.

Index	TLH (n=85)	TAH (n=95)	P
Educational background of the patients			0.529
Primary school	9 (10.6%)	14 (14.7%)	
Middle school	38 (44.7%)	51 (53.7%)	
High school	22 (25.9%)	20 (21.1%)	
College and above	8 (9.4%)	6 (9.5%)	
Educational background of the spouses			0.513
Primary school	3 (3.5%)	5 (5.3%)	
Middle school	29 (34.1%)	43 (45.3%)	
High school	35 (41.2%)	35 (36.8%)	
College and above	10 (11.8%)	8 (8.4%)	

Data are reported as n (%). Statistical analysis was performed with the chi-squared test. TAH: total transabdominal hysterectomy; TLH: total laparoscopic hysterectomy.

**Table 4:** Comparison of overall satisfaction with postoperative sex life between groups.

Index	TLH (n=85)	TAH (n=95)	P
Resumption of sexual activity	77 (90.6%)	91 (95.8%)	0.160
Time to resumption of sexual activity (d)	134.4±56.7	140.1±67.4	0.558
Overall satisfaction			0.128
Increased	0	0	
Unchanged	25 (32.5%)	40 (44.0%)	
Decreased	52 (67.5%)	51 (56.0%)	

Data are reported as means±SD or n (%). Statistical analysis was performed with the chi-squared test. TAH: total transabdominal hysterectomy; TLH: total laparoscopic hysterectomy.

**Table 5:** Comparison of indexes related to postoperative sexual function between groups.

Index	TLH (n=85)	TAH (n=95)	P
Postoperative sexual frequency			0.060
Increased	0	0	
Unchanged	23 (29.9%)	40 (44.0%)	
Decreased	54 (70.1%)	51 (56.0%)	
Postoperative libido			0.128
Increased	0	0	
Unchanged	25 (32.5%)	40 (44.0%)	
Decreased	52 (67.5%)	51 (56.0%)	
Postoperative orgasm			1.000
Increased	0	0	
Unchanged	44 (57.1%)	52 (57.1%)	
Decreased	33 (42.9%)	39 (42.9%)	
Postoperative sexual disorders			1.000
Increased	1 (1.3%)	1 (1.1%)	
Unchanged	76 (98.7%)	90 (98.9%)	
Decreased	0	0	
Postoperative dyspareunia			0.189
Increased	17 (22.1%)	13 (14.3%)	
Unchanged	60 (77.9%)	78 (85.7%)	
Decreased	0	0	

Data are reported as n (%). Statistical analysis was performed with the chi-squared test. TAH: total transabdominal hysterectomy; TLH: total laparoscopic hysterectomy.

## Discussion

The main findings of the present study were that substantial numbers of patients in the TLH and TAH groups reported decreased satisfaction with their sexual life, reduced frequency of sexual activity, decreased libido, orgasm dysfunction, and increased dyspareunia. However, there was no significant difference between groups in any of the indexes of sexual function. Taken together, our preliminary data indicated that TLH and TAH have comparable negative effects on sexual function in women in China treated for benign uterine tumors.

Hysterectomy alters the anatomic relationships, innervation, and blood supply of the pelvic floor, which could theoretically alter sexual function (29). Although several previous studies have suggested that sexual function is often improved after hysterectomy for benign uterine disease due to alleviation of symptoms (10–16), a large minority of patients with benign disorders of the uterus exhibit a worsening of sexual function after hysterectomy (17–19). Indeed, Goktas et al. observed that total hysterectomy and bilateral salpingo-oophorectomy in women with benign disease led to a deterioration in sexual function, which was assessed using the Female Sexual Function Index (20). Gütl et al. (30) also reported the occurrence of sexual dysfunction after hysterectomy, but libido and sexual satisfaction subsequently improved 3 months to 2 years after surgery.

A notable finding of our study was that postoperative sexual function was comparable between the TLH and TAH groups. Our results are consistent with those reported in a small number of previous studies that directly compared laparoscopic and transabdominal hysterectomy in women with benign (31–35) or malignant (36,37) uterine disease. Our analysis identified a higher incidence of sexual dysfunction after hysterectomy than some previous studies (10–16). However, it should be noted that the quality of female sexual life before and after hysterectomy might be affected by numerous factors such as age, ethnicity, family structure, psychologic factors (including body esteem and relationship quality), socioeconomic status and educational background (20,22 [Bibr B23] [Bibr B24] [Bibr B25],^38^,^39^). After surgery, some patients may decrease the frequency of sexual activity due to worry about the adverse effects of sex life. Furthermore, pre-existing mental illnesses such as depression and sexual dysfunction are closely related to postoperative sexual dysfunction (9).

This study had some limitations. First, this was a retrospective analysis and so may have been prone to selection bias or information bias. Second, this was a single-center study, hence the generalizability of the findings is not known. Third, the sample size was small, so the study may have been underpowered to detect some real differences between groups. Fourth, psychologic factors were not assessed before and after surgery, so their possible influence on the results cannot be determined. Additional, prospective, multi-center studies with larger sample sizes are needed to confirm our findings.

In conclusion, our preliminary data suggested that TLH and TAH had similar negative effects on sexual function in women in China treated for benign uterine tumors.

## References

[1] Merrill RM (2008). Hysterectomy surveillance in the United States, 1997 through 2005. Med Sci Monit.

[B02] Hill EL, Graham ML, Shelley JM (2010). Hysterectomy trends in Australia - between 2000/01 and 2004/05. Aust N Z J Obstet Gynaecol.

[B03] OECD Geographic variations in health care: what do we know and what can be done to improve health system performance?. OECD health policy studies.

[4] Liu F, Pan Y, Liang Y, Zhang C, Deng Q, Li X (2017). The epidemiological profile of hysterectomy in rural Chinese women: a population-based study. BMJ Open.

[5] Bretschneider CE, Jallad K, Paraiso MFR (2017). Minimally invasive hysterectomy for benign indications: an update. Minerva Ginecol.

[6] Backes FJ, Fowler JM (2014). Hysterectomy for the treatment of gynecologic malignancy. Clin Obstet Gynecol.

[7] Ramdhan RC, Loukas M, Tubbs RS (2017). Anatomical complications of hysterectomy: a review. Clin Anat.

[8] Danesh M, Hamzehgardeshi Z, Moosazadeh M, Shabani-Asrami F (2015). The effect of hysterectomy on women's sexual function: a narrative review. Med Arch.

[9] Lonnee-Hoffmann R, Pinas I (2014). Effects of hysterectomy on sexual function. Curr Sex Health Rep.

[10] Doganay M, Kokanali D, Kokanali MK, Cavkaytar S, Aksakal OS (2019). Comparison of female sexual function in women who underwent abdominal or vaginal hysterectomy with or without bilateral salpingo-oophorectomy. J Gynecol Obstet Hum Reprod.

[B11] van Zanten F, Brem C, Lenters E, Broeders IAMJ, Schraffordt Koops SE (2018). Sexual function after robot-assisted prolapse surgery: a prospective study. Int Urogynecol J.

[B12] Schiavi MC, Savone D, Di Mascio D, Di Tucci C, Perniola G, Zullo MA (2018). Long-term experience of vaginal vault prolapse prevention at hysterectomy time by modified McCall culdoplasty or Shull suspension: Clinical, sexual and quality of life assessment after surgical intervention. Eur J Obstet Gynecol Reprod Biol.

[B13] Radosa JC, Meyberg-Solomayer G, Kastl C, Radosa CG, Mavrova R, Graber S (2014). Influences of different hysterectomy techniques on patients' postoperative sexual function and quality of life. J Sex Med.

[B14] Costantini E, Porena M, Lazzeri M, Mearini L, Bini V, Zucchi A (2013). Changes in female sexual function after pelvic organ prolapse repair: role of hysterectomy. Int Urogynecol J.

[15] Ge P, Vaucel E, Jarnoux M, Dessaux N, Lopes P (2015). Study of the sexuality of women after a total hysterectomy versus subtotal hysterectomy by laparoscopy in Nantes CHU.[ in French]. Gynecol Obstet Fertil.

[16] Brito LG, Pouwels NS, Einarsson JI (2014). Sexual function after hysterectomy and myomectomy. Surg Technol Int.

[17] Thakar R (2015). Is the uterus a sexual organ? Sexual function following hysterectomy. Sex Med Rev.

[B18] Ucar MG, Ilhan TT, Sanlikan F, Celik C (2016). Sexual functioning before and after vaginal hysterectomy to treat pelvic organ prolapse and the effects of vaginal cuff closure techniques: a prospective randomised study. Eur J Obstet Gynecol Reprod Biol.

[19] Tozo IM, Moraes JC, Lima SM, Goncalves N, Auge AP, Rossi LM (2009). Sexuality evaluation in women submitted to hysterectomy for the treatment of uterine leiomyoma. [in Portuguese]. Rev Bras Ginecol Obstet.

[20] Goktas SB, Gun I, Yildiz T, Sakar MN, Caglayan S (2015). The effect of total hysterectomy on sexual function and depression. Pak J Med Sci.

[21] Plotti F, Terranova C, Capriglione S, Crispino S, Li Pomi A, de Cicco Nardone C (2018). Assessment of quality of life and urinary and sexual function after radical hysterectomy in long-term cervical cancer survivors. Int J Gynecol Cancer.

[22] Wang X, Chen C, Liu P, Li W, Wang L, Liu Y (2018). The morbidity of sexual dysfunction of 125 Chinese women following different types of radical hysterectomy for gynaecological malignancies. Arch Gynecol Obstet.

[B23] Zhou W, Yang X, Dai Y, Wu Q, He G, Yin G (2016). Survey of cervical cancer survivors regarding quality of life and sexual function. J Cancer Res Ther.

[B24] Bogani G, Serati M, Nappi R, Cromi A, di Naro E, Ghezzi F (2014). Nerve-sparing approach reduces sexual dysfunction in patients undergoing laparoscopic radical hysterectomy. J Sex Med.

[B25] Ye S, Yang J, Cao D, Zhu L, Lang J, Chuang LT (2014). Quality of life and sexual function of patients following radical hysterectomy and vaginal extension. J Sex Med.

[26] Pieterse QD, Kenter GG, Maas CP, de Kroon CD, Creutzberg CL, Trimbos JB (2013). Self-reported sexual, bowel and bladder function in cervical cancer patients following different treatment modalities: longitudinal prospective cohort study. Int J Gynecol Cancer.

[27] Cabral PU, Canario AC, Spyrides MH, Uchoa SA, Eleuterio J, Goncalves AK (2013). Determinants of sexual dysfunction among middle-aged women. Int J Gynaecol Obstet.

[28] Mazer NA, Leiblum SR, Rosen RC (2000). The brief index of sexual functioning for women (BISF-W): a new scoring algorithm and comparison of normative and surgically menopausal populations. Menopause.

[29] Celik H, Gurates B, Yavuz A, Nurkalem C, Hanay F, Kavak B (2008). The effect of hysterectomy and bilaterally salpingo-oophorectomy on sexual function in post-menopausal women. Maturitas.

[30] Gütl P, Greimel ER, Roth R, Winter R (2002). Women's sexual behavior, body image and satisfaction with surgical outcomes after hysterectomy: a comparison of vaginal and abdominal surgery. J Psychosom Obstet Gynaecol.

[31] Koroglu N, Aslan Cetin B, Akca A, Turan G, Temel Yuksel I, Safak Yildirim I (2018). A comparison of pelvic organ prolapse and sexual function after abdominal and laparoscopic hysterectomy. Ginekol Pol.

[B32] Kayatas S, Ozkaya E, Api M, Cikman S, Gurbuz A, Eser A (2017). Comparison of libido, Female Sexual Function Index, and Arizona scores in women who underwent laparoscopic or conventional abdominal hysterectomy. Turk J Obstet Gynecol.

[B33] Kurek Eken M, Ilhan G, Temizkan O, Celik EE, Herkiloglu D, Karateke A (2016). The impact of abdominal and laparoscopic hysterectomies on women's sexuality and psychological condition. Turk J Obstet Gynecol.

[B34] Ercan O, Ozer A, Kostu B, Bakacak M, Kiran G, Avci F (2016). Comparison of postoperative vaginal length and sexual function after abdominal, vaginal, and laparoscopic hysterectomy. Int J Gynaecol Obstet.

[35] Lermann J, Haberle L, Merk S, Henglein K, Beckmann MW, Mueller A (2013). Comparison of prevalence of hypoactive sexual desire disorder (HSDD) in women after five different hysterectomy procedures. Eur J Obstet Gynecol Reprod Biol.

[36] Xiao M, Gao H, Bai H, Zhang Z (2016). Quality of life and sexuality in disease-free survivors of cervical cancer after radical hysterectomy alone: a comparison between total laparoscopy and laparotomy. Medicine (Baltimore).

[37] Serati M, Salvatore S, Uccella S, Laterza RM, Cromi A, Ghezzi F (2009). Sexual function after radical hysterectomy for early-stage cervical cancer: is there a difference between laparoscopy and laparotomy?. J Sex Med.

[38] Laumann EO, Paik A, Rosen RC (1999). Sexual dysfunction in the United States: prevalence and predictors. JAMA.

[39] Peterson ZD, Rothenberg JM, Bilbrey S, Heiman JR (2010). Sexual functioning following elective hysterectomy: the role of surgical and psychosocial variables. J Sex Res.

